# On Cerebral Hæmorrhage in the Aged

**Published:** 1891-12-05

**Authors:** 


### THE PRACTITIONER'S MIRROR AND RETROSPECT.

[*The Editor will be glad to receive offers of co-operation and contributions from members of the profession. All letters should be addressed to* THE EDITOR, THE LODGE, PORCHESTER SQUARE, LONDON, W.]

### MEDICINE.

### ON CEREBRAL HÆMORRHAGE IN THE AGED.

Dr. Richardson has somewhere said that we ought to look forward to a time when ordinary diseases shall be extinguished by the steady growth and careful application of the medical art in its wider sense, when life will flow on gently to a natural and painless end, a sort of falling asleep, while the mental and most of the physical powers will be retained till the last, and death itself will be only the last of a series of physiological changes continued from birth. Now it is exceedingly useful to have a perfect ideal set before us, even though we do not always see how it may be completely attained, and to consider the gaps where our knowledge and art fail us, to collect, as Bacon says, the facts which are most necessary to be discovered. Medicine, indeed, has made some little progress towards its ideal, and when the zymotic and infective diseases are reduced by bacteriology, it will have made a great deal more. Among other deficiencies, it needs some better means of preventing one of the greatest horrors of advanced years, the danger of the sudden stroke of paralysis from cerebral hæmorrhage, and the crippled degraded life that follows in such a large number of those who have escaped the storms of earlier days. What are the causes which tend to produce so many instances of cerebral hæmorrhage, and how far is it possible to prevent it? Sir George Humphry has pointed out that the aged are free from many of the scourges which afflict their younger friends, so that if two or three classes of diseases could be eliminated we should look forward almost eagerly to the years of old age as a sweet haven of security where we might spend cheerful days without dread of pain. The old are comparatively exempt from fevers, from tubercular diseases, from those connected with the multiplication of the species, and from a large number of nervous complaints. They are little affected by cancer, and generally lose the dyspepsia which tormented their days of activity. Their wounds and fractures are repaired as quickly and probably with less liability to septicæmia than in younger men. Their troubles are chiefly the remains of former diseases resulting in chronic heart and kidney, bronchial, and rheumatic complaints, but in the prevention and treatment of these the medical art is slowly, yet surely acquiring more and more power. It is true, indeed, that their liability to diseases of cardiac, nervous, and renal origin has increased in England to some extent in the last thirty years, but they still enjoy a considerable degree of immunity. One of their greatest risks is that of cerebral hæmorrhage, and the consequent paralysis and softening of the brain. Since the researches of Charcot and Bouchard this hæmorrhage has been generally ascribed to the effects of miliary aneurisms on small arteries in the majority of instances. Charcot described them as commencing by a periarteritis and an infiltration of the adventitia, after which an atrophy of the muscular coat ensues. Atheroma may or may not be present. It is not directly the cause of them, and, indeed, does not occur in the small vessels on which they are seated, though by hindering the expansion and contraction of the large vessels it may in some cases increase the strain on a weakened vessel and predispose to aneurism. It is remarkable that while these small aneurisms appear to begin by a change in the adventitia, calcareous degeneration has its seat first in the nuclei of the muscular coat, atheroma in the inner coat, and fatty degeneration in the lining of the inner coat itself. So that we have a special form of decay peculiar to each part of the arterial walls.

According to later observers it does not seem that these miliary aneurisms are always present. Possibly rupture may occur without dilation as soon as the degenerative process has advanced far enough. Certainly it may happen from various other diseases of the vessels, such as syphilis, and in the combined high tension and vascular degeneration of certain forms of Bright's diseases. But putting these on one side, what is the cause of the process described by Charcot? Is there an eroding influence from without, since the mischief appears to commence on the outside? It is not enough to point to the loss of support from the wasting of the brain tissues with age, and, indeed, the liability to aneurism seems to decrease slightly in extreme old age after eighty; nor to the general senile atrophy of the tissues. If the walls are weaker, so is the force of the heart less than in youth. There appears to be a localised, not a general change, a loss here and there of "the contractile and elastic elements of the coats," an atrophy of the muscular coat above all. Now if we reflect on the conditions of the cerebral circulation as expounded by Moxon and others, the replacing of wasting tissues by an increase of perivascular fluid, the fact that cerebral anæmia is the most common condition of failing years, and lastly on the mode of nutrition of the arterial coats by vessels which ramify almost entirely in the adventitia, we see at once the cause of Charcot's infiltration of the outer coat and the atrophy of the muscular one in small arteries. The break-down is not due to loss of support or high tension (in this class of cases) or to periarteritis or endarteritis, but to failure of blood supply from the minute nutrient arteries. The elastic tissue, which they should nourish, is replaced by degraded forms such as appear when stagnation and venous blood replace the normal supply, as the nutrient vessels one by one fail to bring it. In the low pressure of the smallest vessels and capillaries the result would be less important, but here pressure soon breaks down the inelastic tissue. The muscular coat too atrophies, perhaps we may say, starved to death without calcareous or other disease, and dilation follows. In large arteries the greater nutricent vessels afford less chance of starvation for the outer coats which we have seen are the ones to fail first.

Thus the aneurisms occur on almost the smallest vessels where pressure and malnutrition are both present. On the smallest and on the large vessels they are not found, for the two conditions do not co-exist. Again, it is chiefly in the brain where the difficulties of maintaining the force of the circulation are greatest, that these aneurisms from starvation appear. A patch of atheroma may give way in any part of the arterial system, starvation of the outer coats only where their food supply depends on arterioles in which the current is reduced to its lowest point. Of course, aneurisms from fatty degeneration and atheroma also occur in cerebral vessels, but their starting-point is different, while Charcot's degeneration is just what the conditions of the cerebral vessels in age would lead us to expect as a frequent occurrence.

Now, in states of high tension we find curiously enough the anæmic condition of old age repeated. Beyond the constriction there is too little blood, and the smaller vessels are starved. Hence, these aneurisms may occur at any age in the high tension of kidney disease. Gowers noticed miliary aneurisms in the retina of a woman of thirty six, who died soon after from cerebral hæmorrhage. Her brain circulation was assimilated to that of an aged woman, because the blood was dammed up. Cardiac hypertrophy is, of course, an additional risk, but only when the contraction of the vessels exists. It is doubtful, Gowers remarks, whether high pressure without diseased vessels ever results in rupture, and in this view we entirely concur. However, in a large proportion of cases in the aged there never has been either cardiac hypertrophy or high tension, but rather the opposite. Our hope in treatment for them must be in improving the nutrition and the cardiac force, and so curing the anæmia. Tonics, iron and arsenic, food, and even regular gentle exercise may do much to prevent the destruction of the vessel walls. When, on the other hand, we have high tension from gout or kidney disease, though extra-arterial anæmia has equally to be combatted, we must reduce the constriction by iodides, nitrites, by elimination of morbid matter, and often reduce the cardiac force too. The indication is sometimes just the opposite of what it would be in the typical aneurisms of the aged. We must not, however, overlook the risk of a variable tension. Dr. Haigh has shown that in some forms of what he terms uric acid-æmia a few doses of acid or alkali will alter the tension entirely, and if we watch minutely a gouty, or perhaps a merely dyspeptic patient with a soft pulse, sudden great rises of pressure will be found to follow some change in the acidity of the urine. These temporary rises of pressure are doubtless a source of great danger. We have not space to discuss whether a sudden fall of pressure may not cause the rupture of diseased vessels as frequently as a rise, though there are grounds for believing this to be the case. Thus hæmorrhage sometimes follows upon the patients taking the erect posture. Sudden changes are certainly to be avoided, and in relation to Dr. Haigh's point we may consider the desirability of a special dietary for patients in whom we have ever noticed much variation of pressure.

Since Charcot's degeneration differs entirely from atheroma and calcareous changes, we see clearly that we cannot diagnose it by the presence of an arcus senilis, or "ossified" arteries, and this is a result of great practical importance; when symptoms of cerebral anæmia exist there is always a danger of the formation of these aneurisms, a danger that demands a perfectly definite treatment. Nor is treatment so hopeless as in the case of atheroma, though probably far more perish from the aneurisms of starvation than from all other senile degenerations put together.

